# Inactivation of non-proteolytic *Clostridium botulinum* type E in low-acid foods and phosphate buffer by heat and pressure

**DOI:** 10.1371/journal.pone.0200102

**Published:** 2018-07-03

**Authors:** Maximilian B. Maier, Tobias Schweiger, Christian A. Lenz, Rudi F. Vogel

**Affiliations:** Lehrstuhl für Technische Mikrobiologie, Technische Universität München, Freising, Germany; University of Connecticut, UNITED STATES

## Abstract

The effect of high pressure thermal (HPT) treatments on the inactivation of spores of non-proteolytic type E *Clostridium botulinum* TMW 2.990 was investigated at high pressures (300 to 600 MPa) and elevated temperatures (80 to 100 °C) in four low-acid foods (steamed sole, green peas with ham, vegetable soup, braised veal) and imidazole phosphate buffer (IPB). In addition, corresponding conventional thermal treatments at ambient pressure were performed to expose possible synergisms of pressure and temperature on spore inactivation. In general, spore count reduction was more efficient by combining pressure and temperatures < 100 °C and the overall process duration could be shortened due to accelerated heating rates (adiabatic effect). Processing at 90 °C and 600 MPa resulted in inactivation below the detection limit after 5 min in all foods except steamed sole. Traditional thermal processing of spores at 90 °C for 10 min, on the other hand, did not result in an estimated 6-log reduction. Additional HPT treatments in steamed sole and IPB did not reveal pronounced food matrix dependent protective effects. Here, varying pressure levels did not appear to be the driving force for spore count reduction in steamed sole at any temperature. By applying a Weibull distribution on destruction kinetics of isobaric/isothermal holding times, 6D-values were calculated. Compression and decompression phase (1 s pressure holding time) had a considerable impact on spore count reduction (max. -2.9 log units) in both, foods and buffer. Hence, compression and decompression phases should directly be included into the total lethal effect of HPT treatments to avoid prolonged holding times and overprocessing.

## Introduction

Non-proteolytic *Clostridium* (*C*.) *botulinum* type E are anaerobic spore-forming bacteria (one out of four phylogenetically different groups) with the ability to produce a highly human-toxigenic neurotoxin [1]. In response to unfavorable conditions (e.g. lack of nutrients or moisture), the vegetative cell can initiate sporulation and transform into an extremely resistant and dormant state. The formed spores are structured in multiple layers (spore coat, outer membrane, cortex, inner membrane and core) that contribute to withstand stress factors such as heat, radiation, high pressures or pH extremes [2, 3]. In contrast to proteolytic strains, spores from *C*. *botulinum* type E strains are able to germinate and grow under refrigerated temperatures as low as 3 °C. Thereby, it is important to note that growth concomitantly may result in toxin production [1] [4] [5]. This hazard is of special concern regarding the microbiological safety of chilled storage foods, especially in the absence of additional hurdles such as pH ≤ 5.0 or water activity ≤ 0.97 [1]. In this scenario, a safe shelf life of chilled foods would solely rely on the preservation process that leads to spore destruction. In general, a thermal treatment at 90 °C for 10 min or equivalent lethality is recommended to achieve a 6-log cycle reduction of non-proteolytic *C*. *botulinum* spores. Such a treatment should ensure proper food safety for up to 10 days at T ≤ 5 °C, provided that a constant and adequate cooling chain is maintained [1] [6] [7]. However, heat sensitive food components, e.g. sea foods or vegetables, can suffer a severe loss in visual, textural and nutritional properties due to thermal processing. These negative effects on food quality can primarily be attributed to low heating rates at ambient pressure, which unavoidably result in higher thermal loads applied on the food product [8]. A potential technology to shorten the total process duration and, simultaneously, inactivate microbial spores is the combination of high pressure and high temperature. High pressure thermal (HPT) processing induces adiabatic heating and cooling caused by physical compression and decompression work, respectively. Adiabatic effects lead to highly homogeneous and rapid temperature changes within the food product and, thereby, constitute a main advantage of this technology [9]. Spore destruction at high pressures (approx. 200 up to 600 MPa) and temperatures (above 60 to 70 °C) is commonly thought to underlie an at least two-step mechanism, which has been described for species of *Bacillus* and *Clostridium*. Initially, sublethally injured spores start to release dipicolinic acid (DPA) due to increasing pressures and temperatures. Accumulated DPA (~20% of spore core dry weight) in the spore’s core decreases the water content and thereby contributes to wet heat resistance. Hence, a DPA release results in partial core hydration and in a concomitant loss of heat resistance. Thereby, DPA release is usually accelerating with increasing process intensity whereat the spore’s resistance is determined by its ability to retain DPA as well as the pressure/heat resistance of the DPA-free spore [2, 3, 10–12]. To date, several studies investigated the HPT inactivation of *C*. *botulinum* type E spores, predominantly focusing on aqueous buffer systems as surrounding matrix [13–20]. Hence, available HPT data on type E spores suspended in relevant food matrices is still scarce. To contribute filling this gap, the present study evaluated the impact of an industrially feasible and preferential pressure range (300 to 600 MPa) in combination with elevated temperatures (80 to 100 °C) on spores of *C*. *botulinum* type E suspended in four low-acid foods and pressure/temperature-stable imidazole/phosphate buffer (IPB). In addition, conventional thermal treatments at ambient pressure served as reference processes in order to evaluate the effect of adding pressure on spore inactivation. Furthermore, a Weibull distribution was applied to fit isobaric/isothermal inactivation curves and, thereby, generate corresponding 6D values. The obtained destruction kinetics can help to reveal possible process parameter combinations for an industrial implementation.

## Materials and methods

### Microorganism, growth conditions and spore production

The non-proteolytic *C*. *botulinum* type E strain TMW 2.990 was used in this study. This strain was chosen for HPT studies because its spores exhibited increased pressure resistance compared to other strains of *C*. *botulinum* type E [20] [18]. Growth conditions and spore purification were performed as described previously [20] [21]. Briefly, spore production started with inoculating 45 mL of tryptone-peptone-yeast extract-carbohydrates (4 g/L glucose, 1 g/L maltose, 1 g/L starch, 1 g/L cellobiose) (TPYC) broth [22] [20] with a -80 °C glycerol stock culture and subsequent incubation in an anaerobic chamber (85% N2, 10% CO2, 5% H2) for 24 h at 28 °C. The growing culture was then transferred into 450 mL of fresh TPYC broth and anaerobically incubated for 12 ± 2 d at 28 °C. The produced spores were harvested by centrifugation (10.000 x g, 4 °C, 10 min), washed three times with ice-cold deionized water and one time with S+ (0.85% saline + 0.1% Antifoam B Emulsion (Dow Corning, Germany)) to reduce possible spore agglomeration, which was followed by incubation in 50% ethanol for 2 h at room temperature. Afterwards, the spore suspension was washed for three more times with ice-cold deionized water and, finally, resuspended in deionized water or in imidazole/phosphate buffer (IPB, pH 7, 50 mM Na_2_HPO_4_ and 50 mM NaH_2_PO_4_ mixed 1:1 with 50 mM imidazole) obtaining viable spore counts of 108–10^9^ spores/mL. Sporulation resulted in a uniform population of phase bright spores ≥ 90% as determined by phase contrast microscopy (S1 Fig). The spore suspensions were stored at 4 °C until use.

### Sample preparation

Samples for experiments in RTE foods were basically prepared as previously described [21]. Briefly, four different heat-sterilized RTE foods (Table 1) were blended into homogeneous pastes, inoculated with spore suspension (spores suspended in deionized water) and thoroughly mixed with a spatula and by vortexing. For experiments in IPB, spores (suspended in IPB) were homogeneously distributed in IPB. Final spore counts were 10^6^ to 10^7^ spores/g before the heat and pressure treatments. For HPT experiments, the inoculated samples were filled into custom-made PTFE tubes and closed with silicon stoppers that were fastened by screw-caps. For thermal treatments, samples were prepared in the same manner, but they were filled into custom-made stainless steel tubes, which were of the same size as the PTFE tubes used for HPT treatments. This allowed for more rapid heating and cooling rates. In addition, uninoculated samples were prepared for temperature profile measurements in the geometrical center of a test vial during processing. All samples were stored on ice before and after treatments.

**Table 1:** Food product formulations [21].

Food product	Fat [wt%]	Protein [wt%]	Carbohydrates [wt%]	Salt [wt%]	Fiber [wt%]	pH	a_w_
**Green peas with ham**	12.8	5.1	5.1	1.1	3.9	6.0	0.973
**Steamed sole**	8.6	10.2	5.7	2.9	1.7	6.75	0.979
**Braised veal**	6.2	7.6	6.7	3.7	1.5	6.53	0.973
**Vegetable soup**	2.9	1.2	5.9	1.0	1.3	5.8–6.0	0.98

wt%, percentage by weight

### High pressure equipment and treatments

The HPT equipment consisted of a hand pump-driven high pressure intensifier system (Unipress, Warsaw, Poland). With an inner volume of 8 mL, two samples could be placed inside the high pressure vessel (type MV2-13, Unipress, Warsaw, Poland) at a time. Bis(2-ethylhexyl) sebacate (Nr. 84822; Sigma-Aldrich, USA) served as a pressure transmitting fluid. The double wall high pressure vessel was temperature-controlled by a circulating oil bath (witeg Labortechnik GmbH, Wertheim, Germany) with silicon oil (Sil 180, Fisher Scientific, New Hampshire, USA) as heating fluid. The lid of the high pressure vessel was equipped with a lead-through for a type K thermocouple. To achieve isobaric/isothermal pressure-holding times, the start of compression began at an empirically determined target pressure-, target temperature-, and sample type-dependent temperature. The temperature was monitored in the geometrical center of an uninoculated sample vial directly above the inoculated sample. Applied pressure levels ranged from 0.1 up to 600 MPa, process temperatures from 80 to 100 °C and pressure holding times from 1 to 300 s. The average compression rate was around 6.5 MPa/s and decompression took place in less than 12 s.

Thermal inactivation experiments were performed similar to the HPT experiments with the exceptions that samples were not pressurized and stainless steel sample tubes were used. Thermal treatments were applied at temperatures ranging from 80 to 100 °C and holding times from 1 to 600 s. The average heating rate was around 0.3 °C/s.

**Fig 1:**
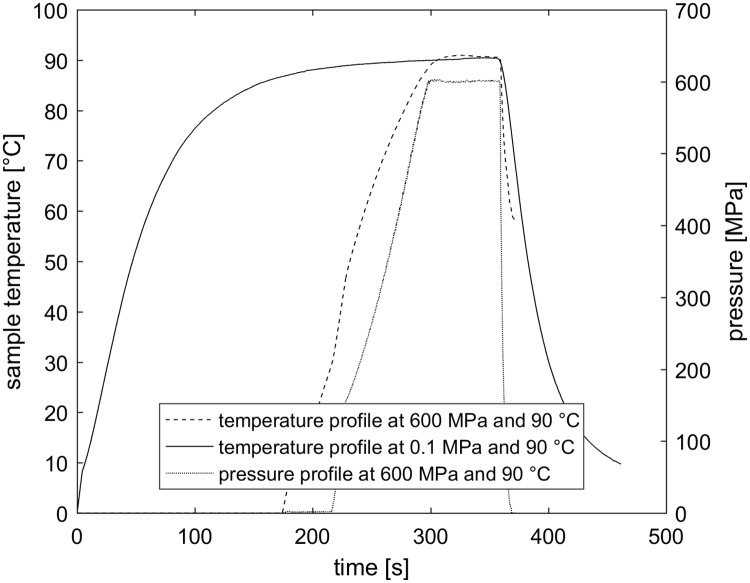
Typical temperature and pressure profiles during HPT and thermal treatments. Exemplarily, the temperature (solid line) profile of a thermal treatment (0.1 MPa, 90 °C) and the pressure (dotted line) and temperature (dashed line) profile of a HPT treatment (600 MPa, 90 °C) with a holding time of 60 s in steamed sole are depicted. HPT heating time: ~124 s. Thermal load of HPT treatment: 12457 °C s. Thermal heating time: ~298 s. Thermal load of thermal treatment: 30766 °C s. The thermal load was calculated via numeric integration of temperature over time.

An exemplary temperature-, pressure- profile is shown in Fig 1. Isothermal holding times were overlapped in order to illustrate differing lengths of heating time. To quantify the total applied thermal load during processing, numeric integration of temperature over time was conducted using MATLAB software (version R2016B, Mathworks, Natick, USA) exemplarily for the shown temperature profiles in Fig 1.

### Enumeration of surviving spores

Surviving spores were enumerated by pour-plating with TPYC agar (15 g/L agar–agar) immediately after treatment. Samples were opened in an anaerobic chamber (85% N_2_, 10% CO_2_, 5% H_2_), serially diluted in S+ solution and pour-plated in duplicate. Survivors were counted after anaerobic incubation for up to 5 d at 28 °C. For visualization, the results are presented as log_10_ (N/N_0_), where N describes the number of surviving spores after a treatment and N_0_ is the initial spore count. The detection limit was defined at a minimum of two colony-forming units in relation to the initial cell count N_0_.

### Statistical analysis and curve fitting

All experiments were conducted at least in independent triplicates. The significance of differences between mean values from independent experiments was determined by one-way ANOVA. Tukey’s HSD test at an error probability of 5% (P < 0.05) served as a post-hoc analysis. Isobaric/isothermal inactivation curves, i.e. excluding the lethal effect of the pressure ramp (1s pressure holding time), were described by applying the following modified Weibull distribution [23]:

$$log\frac{N}{N_{0}}=-{\left(\frac{t}{\delta }\right)}^{ẞ}$$ (1)

or

$$t=\delta {\left(log\frac{N}{N_{0}}\right)}^{\frac{1}{ẞ}}$$ (2)

where *log N/N*_*0*_ is the decimal reduction ratio at a time *t*. The scale parameter *δ* can be considered as an equivalent to the traditional *D* value (first-order kinetic) and represents the time of first decimal reduction. The factor *ẞ* describes the curve shape, where values < 1 imply upward concave (tailing) inactivation patterns and values > 1 describe downward concave (shoulder) curve shapes, and curve linearity is described by *ẞ* = 1 [23, 24]. The flattening of biphasic survivor curves or in other words, drastically decreased inactivation rates at longer holding times are generally described as tailing. The exact reasons remain unclear but proposed contributing factors include heterogeneous resistance properties within a spore population, spore clumping, adhesion to any surfaces during sample handling and protective effects of dead spores [25–29]. In contrast, an initial lag phase or shoulder effect in inactivation curves describes an initial increase in cell count from treated compared to untreated spore samples. The disassembly of spore agglomerates or a pressure-induced germination of superdormant spores are two reasons often discussed in literature [11, 30, 31]. Statistical analysis and curve fitting were performed using MATLAB software (version R2016B, Mathworks, Natick, USA).

## Results

### HPT inactivation in four low-acid foods at 90 °C and 600 MPa

**Fig 2:**
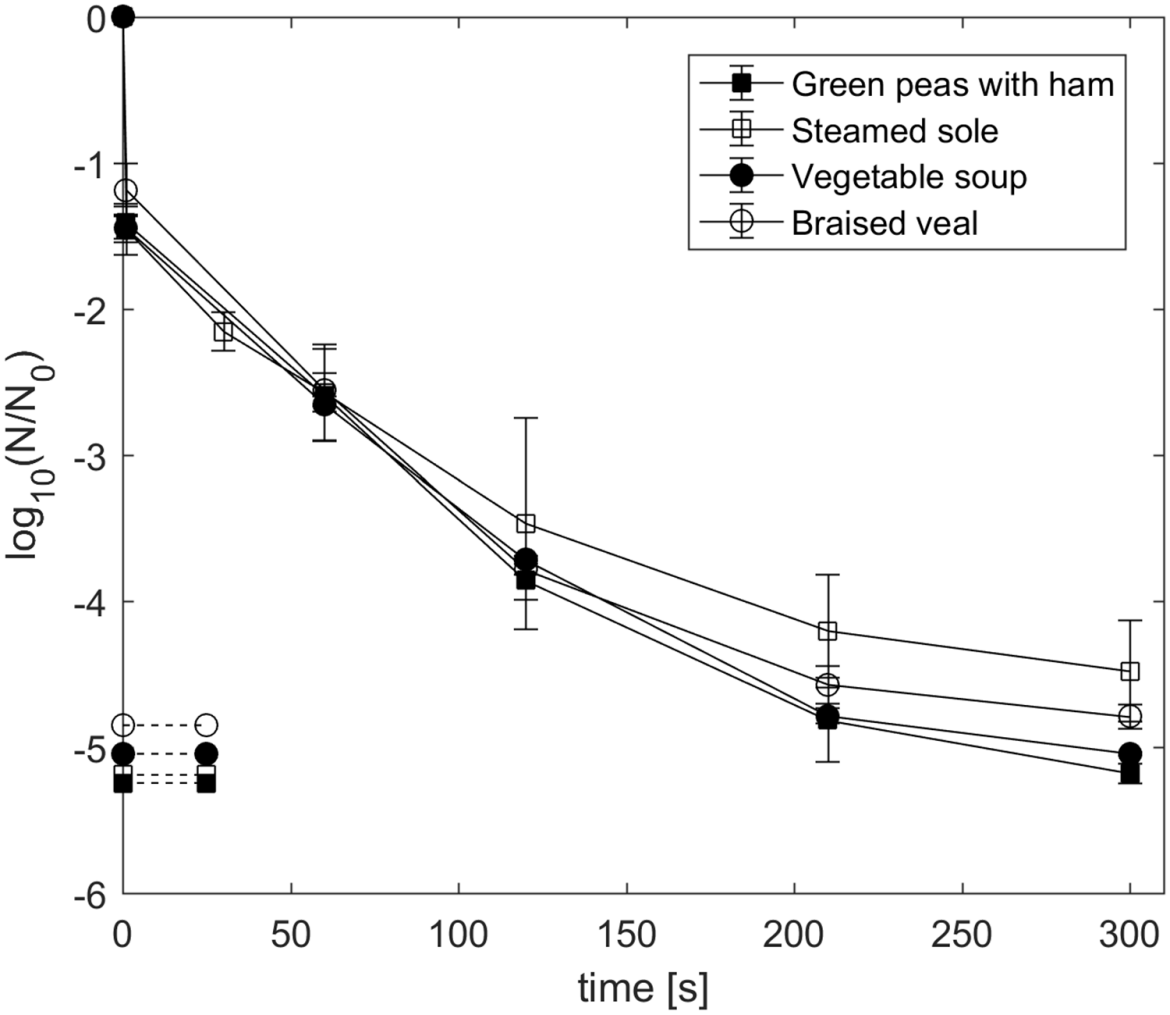
Log reduction of *C*. *botulinum* TMW 2.990. Spores suspended in green peas with ham (solid squares), steamed sole (open squares), vegetable soup (solid circles) and braised veal (open circles) after HPT treatment at 600 MPa and 90 °C for 1 to 300 s. Dashed lines indicate the corresponding detection limits. Initial spore count: 10^6^–10^7^ spores/g. Data are shown as means ± standard deviations of at least three independent experiments.

In general, HPT treatments at 600 MPa and 90 °C resulted in similar spore inactivation in all four foods (Fig 2). Spore inactivation due to compression and instant decompression (1 s holding time) were > 1-log cycles in all foods tested. Further pressurization for up to 300 s under isothermal/isobaric conditions increased spore inactivation by at least 3.0 log units. Inactivation levels reached the detection limit in green peas with ham, vegetable soup and braised veal (detection limits: green peas with ham: at -5.2-log cycles; vegetable soup: at -5.0-log cycles; braised veal: at -4.8-log cycles) after the maximum pressure holding time of 300 s. The Weibull function (Eq 1) was applied to describe and fit non-linear inactivation kinetics during isobaric/isothermal holding times, i.e. excluding lethal effects of the pressure ramp. Generated parameters are shown in Table 2. All *ẞ* values were < 1, which reflects the observed upward concave inactivation pattern. Based on the obtained model parameters, the required pressure holding times to achieve a 6-log cycle reduction at 90 °C and 600 MPa were calculated and resulted in values between 9.6 and 14.5 min. Since spores of non-proteolytic *C*. *botulinum* type E strains were more resistant in steamed sole and their habitats are primarily associated with aquatic environments [32, 33], further HPT inactivation experiments were performed using steamed sole as a food sample matrix.

**Table 2:** Spore count reduction due to pressure ramp and kinetic Weibull model parameters for isothermal/isobaric conditions at 600 MPa and 90 °C.

Food product	Pressure ramp reduction[log N/N_0_]	ẞ	δ [s]	R^2^	6D [min]
**Green peas with ham**	-1.4 ± 0.1	0.6271	33.24	0.98	9.6
**Steamed sole**	-1.5 ± 0.2	0.6083	45.59	0.97	14.5
**Braised veal**	-1.2 ± 0.2	0.5396	24.8	0.96	11.4
**Vegetable soup**	-1.5 ± 0.1	0.6196	34.61	0.98	10.4

### HPT inactivation in steamed sole and imidazole phosphate buffer

**Fig 3:**
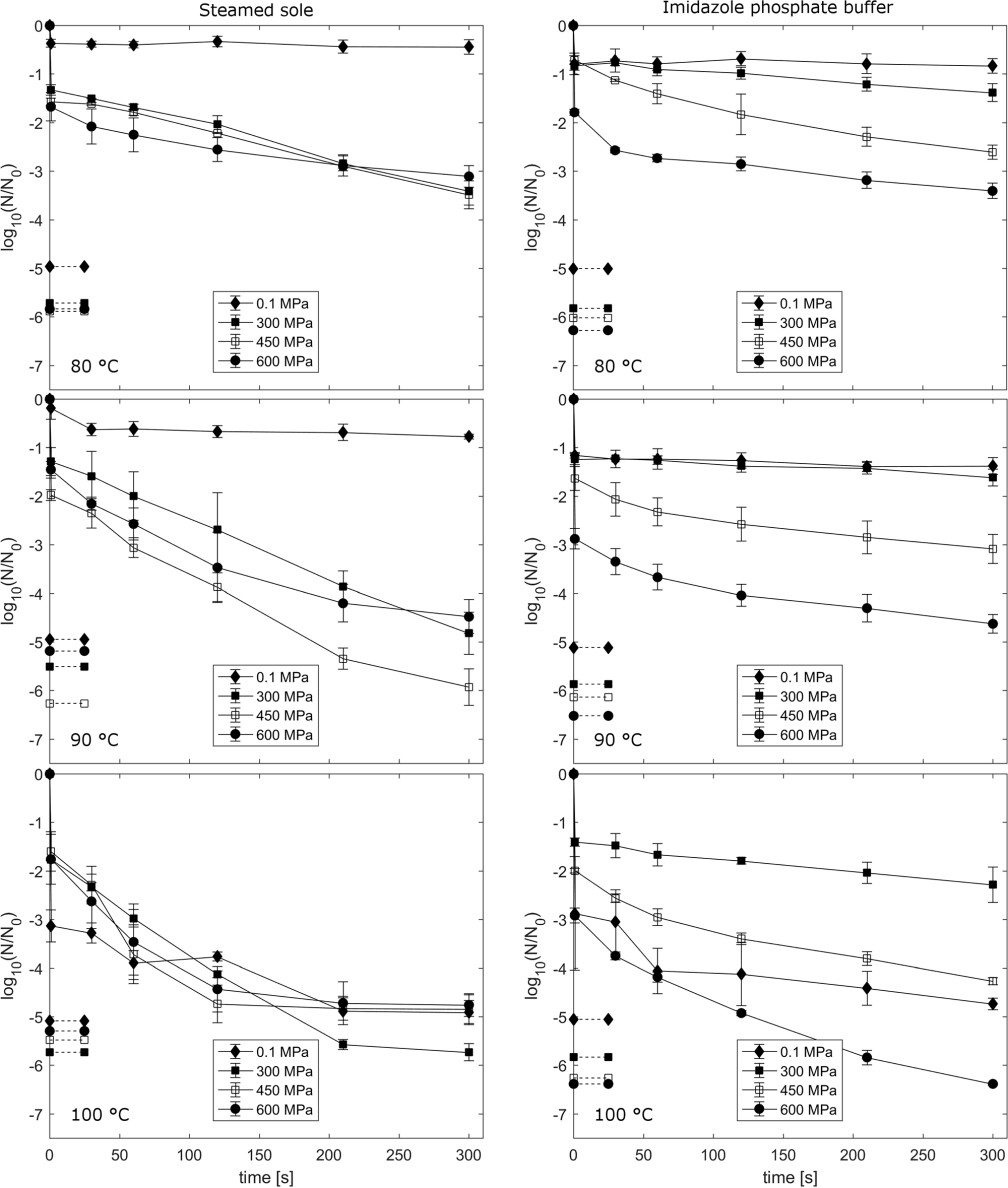
Log reduction of *C*. *botulinum* TMW 2.990. Spores suspended steamed sole and IPB after treatments at 90–100 °C and 0.1–600 MPa for 1–300s. Dashed lines indicate the corresponding detection limits. Initial spore count: 10^6^–10^8^ spores/g. Data are shown as means ± standard deviations of three independent experiments.

Spores of the non-proteolytic type E strain TMW 2.990 were treated in steamed sole and IPB at pressures ranging from 0.1 to 600 MPa and process temperatures of 80 to 100 °C (Fig 3). In general, spore inactivation increased with increasing process intensities in both matrices.

Spores suspended in steamed sole and pressurized at 300, 450 and 600 MPa at 80 °C had similar curve shapes and did not exhibit major differences total inactivation after a maximum holding time of 300 s, which resulted in final log reduction values between 3.1 and 3.5 log units. Thermal treatment at 80 °C and ambient pressure did not result in significant spore count reduction (0.4 log units) after 5 min of holding time. Increasing the process temperature to 90 °C, spores pressurized at 450 MPa exhibited less resistance (5.9 log units) compared to those treated at 300 (4.8 log units) and 600 MPa (4.5 log units). Only a slight inactivation of 0.8-log cycles was achieved by thermal processing at 90 °C for 300 s. Further thermal treatment up to 10 min at 90 °C (not depicted in Fig 3) increased spore inactivation to 1.8 ± 0.1 log units which generated a D_90°C_-value (linear function) of 6.4 minutes. At 100 °C on the other hand, the highest inactivation was achieved by applying a pressure level of 300 MPa, partially reaching the detection limit (-5.7 log units) after 300 s of pressure holding time. Furthermore, treatments at 0.1, 450 and 600 MPa resulted in decelerated spore count reduction after 120 s, approximating the respective detection limit and resulting in similar final inactivation levels.

Regarding the results of HPT treatments of spores suspended in IPB, a more apparent tendency could be observed. At every applied process temperature (80 °C, 90 °C, 100 °C), spore count reduction could be enhanced by increasing the pressure level stepwise from 300 MPa, to 450 MPa and, finally, to 600 MPa. Thermal treatments at ambient pressure generally resulted in an improved spore inactivation with a gradual increase in temperature. At 80 and 90 °C, pressures < 600 MPa were not sufficient to reduce the initial spore count by more than 3.1-log cycles after a maximum holding time of 300 s. Furthermore, thermal treatments at 0.1 MPa and pressurization at 300 MPa resulted in similar spore count reduction (max. 1.6 log units) after 300 s holding time with just marginal benefits of applying pressure. The conventional thermal treatment at 90 °C for 10 min just slightly increased spore count reduction to 1.7 ± 0.2-log cylces (not depicted in Fig 3) compared to 300 s holding time and resulted in a D_90°C_-value of 20.8 min (calculated at isothermal holding time). On the other hand, the final inactivation level was more than doubled by the addition of pressures ≥ 450 MPa compared to thermal treatments at ambient pressure. At 100 °C, the previous described order of inactivation did not fully apply to thermal and HPT treatments. A thermal treatment at ambient pressure was more effective (4.7 log units) than HPT treatments at 300 MPa (2.3 log units) or 450 MPa (4.3 log units). However, the most effective inactivation in IPB was achieved by applying 600 MPa in combination with 100 °C. This treatment resulted in a final log reduction of 6.4 log units, with spore counts falling below the detection limit at the same time.

By comparing the HPT inactivation in both matrices, no definite food matrix-dependent protective effect could be observed for spores suspended in steamed sole in general. Spores tended to be less resistant when pressurized in steamed sole, i.e., embedment in this matrix resulted in predominantly lower or at least similar final spore count reduction. The only exception from this general trend was observed at 600 MPa and 100 °C, where the highest total spore inactivation was achieved in IPB. For process temperatures up to 90 °C, the addition of defined pressure levels clearly accelerated spore inactivation in both matrices. In IPB, pressures ≥ 450 MPa the final inactivation result was at least 2.2-fold higher compared with thermal treatments at ambient pressure. Such a synergistic effect of pressure and temperature was also observed in steamed sole, resulting in at least 5.5-fold higher spore count reduction. Regarding the inactivation effect of the pressure ramp (1 s of pressure holding time), a more pronounced and pressure-dependent impact was observed in IPB, almost reaching a 3-log cycle reduction at 600 MPa and 90 and 100 °C (Tables 3 and 4). Subsequent spore count reduction in IPB during isobaric/isothermal holding times resulted in much flatter curve progressions. Consequently, obtained pressure holding times to achieve a 6-log reduction were generally higher in IPB except for the already mentioned combination of 100 °C and 600 MPa. Based on the calculated model parameters, a desired 6-log reduction within 10 min could be reached in steamed sole at 90 °C with 300 or 450 MPa or at 100 °C in combination with 300 MPa (Table 3).

**Table 3:** Spore count reduction due to pressure ramp and kinetic Weibull model parameters for isothermal/isobaric conditions in steamed sole.

Temperature [°C]	Pressure [MPa]	Pressure ramp/heating phase reduction [log N/N_0_]	ẞ	δ [s]	R^2^	6D [min]
80	0.1	-0.4 ± 0.1	-	-	-	-
80	300	-1.3 ± 0.1	1.0360	153.7	0.99	14.4
80	450	-1.6 ± 0.1	1.1400	178.6	0.99	14.3
80	600	-1.7 ± 0.3	0.5379	160.4	0.99	74.8
90	0.1*	-0.2 ± 0.2	0.777	365.9	0.84	61.2
90	300	-1.3 ± 0.3	0.9107	81.6	0.99	9.7
90	450	-2.0 ± 0.1	0.8108	54.0	0.98	8.2
90	600	-1.6 ± 0.2	0.6524	73.1	0.97	19.0
100	0.1*	-3.1 ± 0.3	0.7980	123.6	0.91	19.5
100	300	-1.8 ± 0.1	0.7193	39.4	0.97	7.9
100	450	-1.9 ± 0.1	0.4111	13.9	0.85	18.0
100	600	-2.3 ± 0.1	0.3854	17.5	0.91	30.4

*Conventional first-order kinetic D values during isothermal holding time were calculated for 90 and 100 °C at ambient pressure: D90°C = 6.4 min; D100°C = 2.5 min.

**Table 4:** Spore count reduction due to pressure ramp and kinetic Weibull model parameters for isothermal/isobaric conditions in IPB.

Temperature [°C]	Pressure [MPa]	Pressure ramp/heating phase reduction [log N/N_0_]	ẞ	δ [s]	R^2^	6D [min]
80	0.1	-0.8 ± 0.2	-	-	-	-
80	300	-0.8 ± 0.1	1.3950	448.7	0.96	27.0
80	450	-0.7 ± 0.1	0.6356	106.3	0.99	29.7
80	600	-1.8 ± 0.1	0.3837	85.7	0.96	152.4
90	0.1*	-1.2 ± 0.1	0.8347	1467.0	0.97	209.2
90	300	-1.2 ± 0.1	1.5350	576.0	0.96	30.8
90	450	-1.6 ± 0.3	0.5076	141.1	0.99	80.2
90	600	-2.9 ± 0.2	0.5291	102.5	0.99	50.5
100	0.1*	-2.9 ± 1.2	0.4969	82.8	0.92	50.8
100	300	-1.4 ± 0.1	0.876	350.5	0.99	45.2
100	450	-2.0 ± 0.1	0.5699	71.0	0.99	27.5
100	600	-2.9 ± 0.2	0.6262	39.9	0.99	11.6

*Conventional first-order kinetic *D* values during isothermal holding time were calculated for 90 and 100 °C at ambient pressure: D_90°C_ = 20.8 min; D_100°C_ = 2.3 min.

## Discussion

The thermal as well as the HPT tolerance of a resistant non-proteolytic *C*. *botulinum* type E strain (TMW 2.990) [18, 20] was determined in four different low-acid foods and in pressure/temperature-stable imidazole/phosphate buffer. All HPT experiments were conducted under isothermal/isobaric conditions to eliminate temperature inconsistencies due to adiabatic heating. Therefore, comparable results among different process parameters (e.g. matrix, target pressure, target temperature) were generated. For an industrial implementation of HPT technology, current maximum feasible and economical pressure levels have to be considered. Therefore the focus of this study was on pressure levels ≤ 600 MPa [34].

Spore inactivation by HPT treatments in all four different foods, which covered a broad range of intrinsic properties and would enable growth of non-proteolytic *C*. *botulinum*, was characterized by similar negative exponential curve shapes with a slightly but significantly (p < 0.05) increased spore resistance in steamed sole (Fig 2). Taking the conventional thermal treatment into account (90 °C, 0.1 MPa, 10 min), in three out of four food matrices, a theoretical 6D spore count reduction was not achieved during isobaric/isothermal holding times within 10 min (based on Weibull parameters; Table 2). However, the heating rate could considerably be increased by applying pressure (approx. 2.4-fold faster) and more importantly the final spore counts fell below the detection limit in three out of four foods (Fig 1). Hence, the addition of pressure substantially increased spore inactivation, shortened the overall process duration and concomitantly reduced the applied thermal load by 59.5%. Furthermore, considerable lethal effects of the compression and decompression phase (> 1-log cycle) were not included into the calculated times for 6D reductions. Therefore, the compression and decompression phase could serve as additional safety margins or directly be integrated into the total lethal effect of HPT treatments. This observed non-negligible impact is in line with previous studies, which investigated the HPT inactivation on spores of several *Clostridium* and *Bacillus* species [21, 26, 29, 35, 36]. Thereby, the impact of varying compression and decompression rates should also be taken into account, since they can largely affect spore inactivation. This has been shown especially for spores of *Bacillus* species, whereby slow pressurization rates enhanced spore count reduction for pressure holding times < 5 min [37] [38]. Typical industrially applied pressurization rates can be up to 3.3 MPa/s [38]. The time to reach certain levels of pressure reported in our study were almost twice as fast. Considering this, finding a balance between sufficient inactivation due to pressurization and economic feasibility with regard to total process time could be an additional challenge for future HPT applications.

Available studies on the HPT inactivation of spores of *C*. *botulinum* type E comprise a broad pressure range between 300 to 1200 MPa but commonly do not exceed temperature levels > 80 °C [13–18, 20]. This can probably be attributed to the fact that spores of non-proteolytic strains of *C*. *botulinum* typically exhibit less resistance towards conventional thermal processing when compared to other food safety relevant spore-forming bacteria [1, 39]. Nevertheless, existing data on thermal inactivation of spores of *C*. *botulinum* type E indicate a broad spectrum of resistance properties. Lindström et al. [40] for instance, reported highly biphasic destruction curves, which resulted in thermal D-values of up to 7.1 min (at 90 °C) for *C*. *botulinum* type E spores suspended in whitefish medium and recovered in medium containing lysozyme, which can possibly assist in germination of heat-stressed spores. Regarding this, in a worst-case scenario, a 6-log cycle reduction would theoretically take 42.6 min of thermal holding time, also meaning, that the recommended 10 min thermal treatment would basically result in a 1.4-log cycle reduction. The fact that properly performed conventional thermal preservation processes are sufficient to ensure consumer safety is putatively mainly related to low contamination levels of *C*. *botulinum* in food samples in general [11]. In our study, we obtained similar spore count reduction values in steamed sole (approx. 1.8 log units) and IPB (approx. 1.7 log units) after 10 min at 90 °C but quite different linear thermal D-values for isothermal holding times. The heating phase in IPB had a much more severe impact on spore inactivation than in steamed sole, which resulted in a smaller curve slope during isothermal holding time and hence, a higher D-value. Temperatures below 90 °C at ambient pressure resulted in marginal spore count reduction (< 1 log units) in both matrices (Fig 2), which indicated insufficient process intensities. Below 100 °C, the addition of pressure clearly enhanced spore inactivation, acting synergistically with heat in steamed sole and partially in IPB (for p > 300 MPa). Such synergisms of pressure and temperature on HPT inactivation for several pathogenic and non-pathogenic spore-formers in various buffer and relevant food systems have previously been reported and are basically in line with our observations [12, 21, 29, 36, 41–43]. However, opposite effects, i.e. pressure-protection at certain p/T combinations, have been reported for spores of proteolytic *C*. *botulinum* [21, 26] and recently, for spores of non-proteolytic *C*. *botulinum* strains [44]. Such pronounced pressure-mediated protective effects (resulting in strong tailing) could be related to a small but highly resistant fraction within a heterogeneous spore population. In general, several factors can contribute to a variability in spore resistance properties and alter the outcome of HPT inactivation studies. Such factors include, among others, sporulation conditions (e.g. medium mineral content, sporulation temperature), spore purification (spore stock purity), spore surrounding matrix (e.g. water content, pH, fat content) process control and intensity (e.g. temperature monitoring, p/T/t conditions) as well as recovery conditions (e.g. incubation time, medium) [11].

In contrast to spores suspended in IPB, varying high pressure levels did not seem to be the driving force for spore inactivation in steamed sole at any applied temperature. In general, spores tended to be less resistant in steamed sole by exhibiting a much more rapid spore inactivation, which was also indicated by shorter treatment times required to obtain a 6D reduction (Tables 3 and 4). In terms of matrix dependent protective effects, Reddy et al. [45] found that non-proteolytic spores of *C*. *botulinum* suspended in crabmeat blend were not protected by the surrounding food matrix in comparison to spores pressurized in phosphate buffer (0.067 M, pH 7). This is basically in agreement with our findings shown in Fig 3 with an exception for HPT experiments conducted at 100 °C and 600 MPa. Here, the overall spore inactivation was lower and the detection limit was not reached for spores suspended in steamed sole. As indicated above, Skinner et al. [44] observed protective effects of non-proteolytic spores of *C*. *botulinum* in ACES buffer (0.05 M, pH 7) due to the addition of high pressures (600 to 700 MPa) to elevated temperatures (83 to 91 °C). In fact, we only observed such a pressure protective effect for p > 300 MPa and, in contrast, solely for spores suspended in steamed sole rather than in IPB. Interestingly, Lenz et al. [18] observed an accelerated DPA-release of spores of TMW 2.990 after treatments at 300 MPa at 30 °C compared to treatments at higher pressure levels (450 MPa, 600 MPa, 750 MPa) at which the spore’s ability to retain DPA may be favored under certain conditions and result in an increased resistance. HPT processing in such defined parameter range could also be related to the spore surrounding food matrix, however, such a complex food matrix did not allow to spot specific responsible food components. So in principle, a protective effect of pressure towards temperature inactivation seems to be identifiable for all spore formers [21, 26, 44], however, the parameter combinations vary and such defined conditions cannot be met in industrial applications of today.

Spore inactivation by HPT obtained in IPB gradually increased with increasing process intensities and are generally supporting and complementing inactivation kinetics reported by Lenz et al. [18] for TMW 2.990 conducted at 30 to 75 °C and 300 to 1200 MPa. By exploiting even higher pressures, it was shown that pressures > 600 MPa noticeably increased spore count reduction. For instance, inactivation results obtained at 75 °C and 750 MPa (approx. -4.5 log units) reached equivalent spore count reduction to those at 90 °C at 600 MPa (-4.6 log units) in our study. However, data reported here demonstrate that a direct transfer of inactivation results obtained in buffer systems to industrial applications, i.e. refrigerated low-acid foods, is difficult, since pressure/temperature dependencies in IPB were not applicable to steamed sole. With almost equal inactivation results at 100 °C and ambient pressure in both matrices, the impact of heat drastically increased and partially obliterated the synergism of pressure and heat. Inactivation during the heating and cooling phase with no holding time reached up to 3-log cycles in both matrices, probably due to slower heating rates involving extended temperature exposure close to 100 °C.

Similar to proteolytic *C*. *botulinum* spores, natural contamination levels of non-proteolytic *C*. *botulinum* spores are normally very low [11]. This and the relatively low effectiveness of the conventional thermal processing conditions that are recommended to inactivate spores from non-proteolytic *C*. *botulinum* strains in a worst-case scenario as described above suggests that HPT processing can serve as an suitable alternative preservation technology to obtain safe chilled foods with extended shelf-life. Possible process parameters for this purpose may be found at p/T/t combinations of 600 MPa, ≥ 90 °C and 300 s (≥ 4.5 log units for all four foods tested) and 300 to 600 MPa, ≥ 80 °C and 300 s (≥ 3 log units for steamed sole). However, taking into account the high variability of spore resistance properties and possible protective p/T combinations, additional safety margins such as strict and constant storage below 3 °C should be considered for low-acid chilled storage products.

## Supporting information

S1 Fig Microscopic picture of purified spore suspension of *Clostridium botulinum* TMW 2.990. (PPTX)
