# Supplementary figures and images for: Inactivation of non-proteolytic Clostridium botulinum type E in low-acid foods and phosphate buffer by heat and pressure
Source: PLoS One. 2018 Jul 3;13(7):e0200102. doi: 10.1371/journal.pone.0200102 (PMC6029780; doi:10.1371/journal.pone.0200102)

## Slide 1
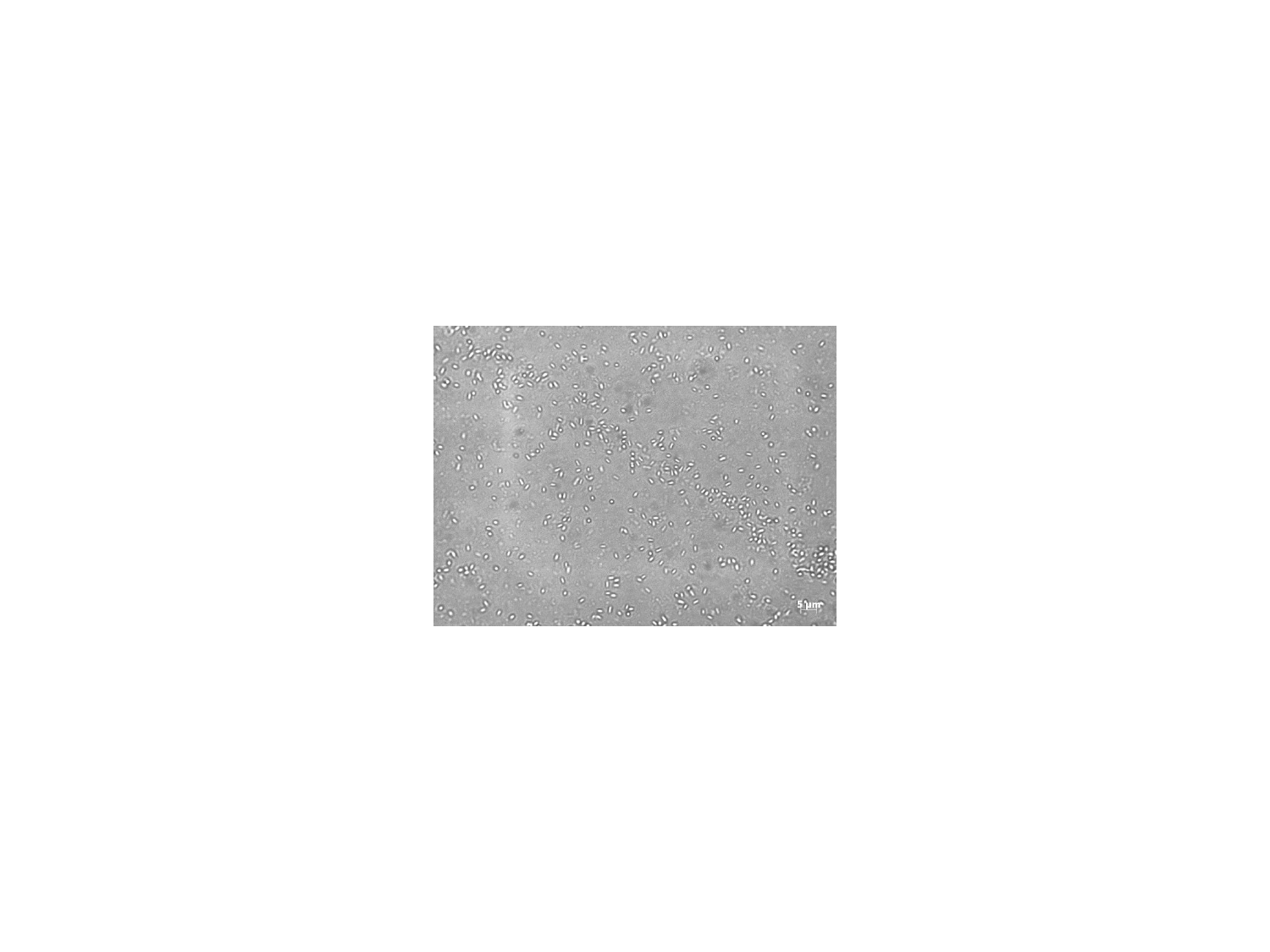

Supplement: S1 Fig — (PPTX) [file pone.0200102.s001.pptx]
